# “One a Day Keeps the Prison Away”: Understanding the Experiences of Individuals Convicted of Sexual Offences Receiving Anti-Androgens for the Treatment of Problematic Sexual Arousal

**DOI:** 10.1007/s10508-024-02847-z

**Published:** 2024-04-09

**Authors:** Rebecca Lievesley, Helen Swaby, Belinda Winder, Christine Norman, Kerensa Hocken

**Affiliations:** 1 NTU Psychology, School of Social Sciences, Nottingham Trent University, 50 Shakespeare Street, Nottingham, NG1 4FQ UK; 2 Midlands Psychology Services, His Majesty’s Prison and Probation Service, Nottingham, UK

**Keywords:** Problematic sexual arousal, Anti-androgens, Pharmacological therapy, Sex offenders

## Abstract

Problematic sexual arousal (PSA) is an umbrella term to describe a range of clinical presentations related to excessive sexual thinking (e.g., sexual preoccupation) and sexual behavior (e.g., hypersexuality). Although such concepts are known to affect sexual recidivism among individuals convicted of sexual offences, PSA is not routinely or directly targeted in offending behavior programs in England and Wales. However, in recent years, there have been moves to incorporate pharmacological interventions for addressing this among people with sexual offence histories. Although some work to understand the experiences of those taking SSRI medication for this purpose has emerged, little is known about the experiences of service users taking anti-androgen medication. In this study, we interviewed all individuals in prison taking anti-androgens for the treatment of problematic sexual arousal following convictions for sexual offences in England at the time of data collection (*N* = 10). Using a phenomenologically oriented thematic analysis, we established themes pertaining to “Differing needs: Motivations for treatment,” “Medication as a risk management strategy,” and how the medication helped the men in their pursuit of “Discovering a ‘new me’.” This work contributes important knowledge to inform the development of ethical and effective prescribing of anti-androgen medication with this population and offer recommendations for both future research and the development of clinical practice.

## Introduction

Issues related to sexual arousal, such as hypersexual behavior and sexual preoccupation, have long been implicated in both first-time sexual offending (Finkelhor, 1984; Seto, 2019; Ward & Beech, 2017) and recidivism among men with sexual convictions (Hanson & Harris, 2000; Hanson & Morton-Bourgon, 2004; Hanson et al., 2007; Knight & Thornton, 2007). Owing to debates within the literature about the precise definitions of pertinent concepts (e.g., hypersexuality, sex addiction, sexual preoccupation and compulsivity; see Walton et al., 2017), we use the label “problematic sexual arousal” (PSA) in this work to capture a range of clinical issues related to excessive, intense, and/or intrusive sexual thoughts, and/or behaviors. Despite being known to predict recidivism, these issues are not uniformly a specific treatment target within offending behavior programs when working with individuals convicted of sexual offences (ICSOs) in England and Wales.^1^ However, in 2007, His Majesty’s Prison and Probation Service (HMPPS) suggested that pharmacological treatment could be used to supplement psychological offending behavior programs after reviewing the promising results of such medications in people who present with sexual disorders and paraphilic sexual interests (Bradford & Kaye, 1999; Briken et al., 2001; Guay, 2009). The HMPPS protocol allows for the use of pharmacological treatments with ICSOs on a voluntary basis (Home Office, 2007), and a three-year pilot trial was commenced in 2007 at HMP Whatton—a therapeutic prison exclusively housing men convicted of sexual offences (Lievesley et al., 2013). This pilot has since been extended and rolled out nationally as a treatment pathway.

This service involves the administration and prescription of two main classes of medication; selective serotonin reuptake-inhibitors (SSRIs; most commonly Fluoxetine) and testosterone-lowering agents, including anti-androgens (most commonly Cyproterone Acetate; CPA) and gonadotropin releasing hormone (GnRH) agonists. According to the prescribing protocols, the choice of medication is determined by the individual’s clinical presentation (Grubin, 2017). That is, those with more psychological issues related to PSA (e.g., sexual preoccupation, in the form of excessive sexual interest or attention) are typically prescribed SSRIs, and those with more prominent behavioral facets of PSA (e.g., compulsive or hypersexual behavior) are prescribed anti-androgens. Although preliminary quantitative evaluations of the effects of these medications (Winder et al., 2014, 2018) and qualitative accounts of the lived experiences of ICSOs taking SSRIs (Lievesley et al., 2014) have been presented elsewhere, this study explores the lived experiences of ICSOs taking anti-androgen medications as a form of medication to manage problematic sexual arousal (MMPSA) in a prison context. These experiences are important to understand, as anti-hormonal medications (such as anti-androgens) are becoming increasingly used as a first-line pharmacological treatment for men with PSA who have committed a sexual offence (Czerny et al., 2002; Turner et al., 2013). However, we do not understand how this medication is experienced (and, subsequently, what this means for treatment maintenance and efficacy).

Anti-androgens are among the most used pharmacological treatments for the reduction in sexual drive with sexually convicted population across the world (Holoyda & Kellaher, 2016; Khan et al., 2015; Ly et al., 2020; Turner et al., 2019), having been used for this purpose since the 1960s (Meyer & Cole, 1997). Anti-androgens work by reducing levels of circulating testosterone, a hormonal steroid found to be associated with sexuality, cognition, personality and aggression (Pfaus, 2009). More specifically, CPA (as the anti-androgen typically used in HMPPS protocols) works by inhibiting the uptake of testosterone, and the release of gonadotropin in the central nervous system, which further reduces testosterone secretion (Jeffcoate et al., 1980; Maletzky & Field, 2003; Pfaus, 2009). Its use with ICSOs and for the reduction in sexual drive has shown positive results, including demonstrable reductions in sexual behavior, libido and sexual arousal, and engagement with sexual fantasies that all may be associated with criminal behavior if acted upon (Amelung et al., 2012; Bradford & Pawlak, 1993; Cooper, 1981; Hoffet, 1968; Landgren et al., 2020; Landgren et al., 2022; Ly et al., 2020; Seebandt, 1968; Winder et al., 2014, 2018). However, these studies are not without their limitations (e.g., they are dated and sparse), leading to a limited evidence base for the effectiveness and suitability of the use of these medications (Briken & Kafka, 2007; Garcia & Thibaut, 2011; Khan et al., 2015). The widespread use of anti-androgens is thus somewhat surprising, especially when considering this limited evidence base in the context of their potentially serious long-term side effects (for a discussion, see Lippi & van Staden, 2017). These include gynecomastia (breast development), weight gain, osteoporosis (reduced bone mineral density), and hepatotoxicity (liver damage caused by pharmaceuticals). In addition, the Medicines and Healthcare Products Regulatory Agency (MHRA) published new evidence that risk of meningioma (tumour growth [usually non-cancerous] from the membrane that surrounds the brain and spinal cord) increases with increasing cumulative dose of CPA (MHRA, 2020). Such effects may influence the extent to which service users are willing to engage or continue with pharmacological treatment, which in turn may impact their levels of risk for reoffending (see Colstrup et al., 2020).

These side-effects, alongside the lack of randomized controlled trials of anti-androgen use for reducing PSA among those who have committed sexual crimes, lead to an ethical dilemma (Çöpür & Çöpür, 2021; Harrison & Rainey, 2009; Ly et al., 2020; Turner et al., 2019), especially in secure forensic contexts wherein some service users need to demonstrate a reduction in risk to become eligible for parole. Without a full understanding of the effects and experiences of receiving anti-androgens for the purpose of reducing PSA, there is a risk of treatment becoming a coercive practice that people with convictions feel obliged to engage with to demonstrate some engagement with available treatment. It is therefore important to understand the experiences of men taking anti-androgens for this purpose to understand their experiences of such side effects as this may have implications for understanding issues such as informed consent and longer-term compliance with medication. Qualitative methods can assist with this, offering an opportunity to tell a story from the service user perspective (Patton, 2002). With the aforementioned ethical concerns in mind (i.e., the limited evidence base in relation to clinical effectiveness in this population and potential side effects), an exploration of service user experiences and perspectives is important.

Although some studies have started to look at the use of anti-androgens in related populations (e.g., those with paraphilic interests in the community; Amelung et al., 2012; Briken et al., 2001), these are usually limited to studies of treatment effects in relation to sexual risk, self-esteem, and sexual activity and thinking (for a review of this literature, see Lewis et al., 2017). This approach has also typically been taken when looking at the use of anti-androgens in convicted groups (Czerny et al., 2002; Turner et al., 2013). To our knowledge, only one other study has qualitatively explored the experience of anti-androgen medication in a sample of men convicted of sexual offences (Boons et al., 2021). This study, which involved the interviewing of patients in a Belgian forensic hospital, offered some insight into the general attitudes towards taking the medication, and documented some service user experiences of anti-androgens (including physical side-effects and broad reductions in sexual thinking). However, there are key differences between Boons et al.’s (2021) work and the current investigation, with the Belgian sample being coerced into taking anti-androgen medication as a requirement of their admittance to the hospital. In contrast, within the English and Welsh context, the prescription of anti-androgens occurs on a voluntary basis. We therefore know very little about the various trajectories both into and through the receipt of MMPSA and considering ICSOs’ motivations for seeking medication and their continued experience of such in the light of the known side effects of anti-androgens.

As such, the study reported in this paper offers a unique and novel contribution to the emergent evidence base related to the use of anti-androgens with ICSOs on a voluntary basis. The significance of such an investigation is in its ability to contextualize the prescribing of anti-androgens in such a way as to inform ethical practice recommendations for treatment guidance and discussions to ensure that those consenting to anti-androgen treatment do so in a fully informed way. This shift in emphasis (from clinical indices of behavior change to a more phenomenological view of the experience of receiving anti-androgen medication) can also bring about a change in how we view the treatment of PSA, from an exclusively risk-based focus to one that places a heavier emphasis on health and wellbeing.

## Method

### Participants

The participant sample consisted of 10 adult males who were receiving anti-androgen medication (CPA; trade name: Androcur) for the purpose of managing their PSA. This represents all individuals taking this medication at the research site at the time of data collection. All participants were serving a prison sentence in a category C prison in England at the time of data collection for a sexual offence (or an offence with a sexual element). All participants were White British with a mean age of 46 years (*SD* = 15.1; range = 24–68), and a mean IQ of 85 (*SD* = 16.3; range = 59–107). Further participant information is detailed in Table 1.

**Table 1:** Participant information

Participant	Index offence(s)	Previous sexual convictions	Daily medication dose (mg)	Length of time on medication
1	Producing, distributing, and possessing indecent images × 2	Yes	50	6 months
2	Sexual assault (child) × 2; Rape (child) × 2	No	50	1.5 years
3	Possessing indecent images	Yes	50	9 weeks
4	Rape (adult)	Yes	50	5 months
5	Rape (child) × 3; Attempted buggery × 2; Rape (adult) × 2; Indecent assault (child) × 3	Yes	100	4 months
6	Indecent assault (adult); Sexual assault (child)	No	100	8 months
7	Arson; Indecent exposure × 4	Yes	50	7 months
8	Assault occasioning actual bodily harm (adult) × 9; Battery (adult) × 2; Sexual assault (child); Possessing indecent images × 7	No	100	4 months
9	Murder	No	50	2 months
10	Producing, distributing, and possessing indecent images × 7	Yes	100	9 months

*Note:*Daily medication dose and length of time on medication correct at the time of data collection

### Procedure

Participants were recruited using a purposive strategy targeting suitable individuals receiving CPA at the treatment site. Potential participants were identified by the prescribing psychiatrist (all those currently receiving CPA), with letters distributed detailing the nature and purpose of the research. It was made clear that participating (or declining) in the research would not affect treatment program selection, medical treatment or parole assessments. All individuals that were contacted (all those receiving CPA) expressed a desire to participate and so written consent was obtained and an interview scheduled for each participant.

The data were collected through semi-structured interviews which took place in purpose-built interview rooms allowing participants the privacy to talk openly about their experiences without being overheard. The interview schedule was designed to allow participants to provide full and comprehensive responses and explored themes related to participants’ offending behavior (e.g., “Can you tell me about how you came to be in prison (your offending)?”), development of PSA (e.g., “Can you tell me about when you first started thinking about sex/sexual behaviors?”, “How have your sexual thoughts/behaviors changed over the years?”), experiences of treatment (including medication (e.g., “Can you tell me about the medication you are taking for your sexual arousal?”)), and their future plans (e.g., “How do you see your future in relation to your sexual arousal”?). A total of 16 interviews were conducted, with 1–2 interviews per participant with each lasting between 50 and173 min (*M* = 100 min). Most participants were interviewed on a one-to-one basis by the lead author with the exception of the two cases where individuals were on high alert status within the prison and could not be interviewed by lone females. In these cases, one of the other authors was also present during the interview. Following each interview, participants were debriefed and provided with information on how to access support should they need it.

### Data Analysis

The interview data were analyzed using a phenomenological approach. Phenomenological methods seek to uncover and understand participants’ subjective experiences and are considered most appropriate when studying topics that are novel, complex and emotionally laden (Smith, 2015; Smith & Osborne, 2003). Thematic analysis is widely recognized for its ability to work with qualitative data in a flexible way to identify, analyze, and interpret patterns (themes) that provide rich accounts of participants’ experiences (Braun & Clarke, 2021). In our phenomenological approach, participants are viewed as the subject “experts,” with the aim of the analysis being to obtain knowledge and insights that stem from their lived experience of being prescribed anti-androgens for the treatment of PSA (for a discussion, see Larkin et al., 2006). In adopting this approach, we attempt to make sense of participants’ sense-making (known as the double hermeneutic; Smith, 2015; Smith & Osborn, 2003).

The analysis was conducted in line with established thematic analysis guidance (Braun & Clarke, 2006, 2021), which involved multiple readings of interview transcripts to increase familiarity with the data while searching for and making note of initial patterns and thoughts. Codes were then assigned to parts of the data considered particularly important or interesting, before these codes were brought together into preliminary themes. The themes were then reviewed and developed, with the meaning and relationship between codes and themes being explored. Within this stage, our phenomenological approach influenced how we achieved this, with interpretations being made about the subjective and lived experiences of participants. Following this, the themes were reviewed and refined to ensure that the analysis reflected the data set as a whole, before assigning names that captured the essence of each theme, and developing a narrative of participants’ lived experiences.

While it is not a requirement of thematic analysis, or qualitative research more broadly, we ensured that our analysis was rigorous by engaging in a form of inter-rater reliability. The lead author (an experienced researcher) conducted the full analysis, with sections of analysis being checked by co-authors to assess the accuracy and validity of the interpretations being made (Lincoln & Guba, 1985; Willig, 2008). All authors are experienced in working with and/or conducting research with individuals with sexual convictions, with one author being a qualified therapist and another being a Principal Psychologist within HMPPS.

## Results and Discussion

Three main themes were derived from participant narratives as being pertinent to their experiences of receiving anti-androgen medication for the treatment of PSA. A summary of these is presented in Table 2.

**Table 2:** Overview of themes

Main themes	Sub themes
1. Differing needs: Motivations for treatment	1.1. Recognizing a need: Intrinsic motivations for treatment
	1.2. Forced compliance: Extrinsic motivations for treatment
2. Medication as a risk management strategy	2.1. “Too risky” without medication
	2.2. “One a day keeps the prison away”
	2.3. A small price to pay
3. Discovering a “new me”	3.1. Adjusting to new sexual norms
	3.2. An awakening

### Theme 1: Differing Needs: Motivations for Treatment

#### Theme 1.1. Recognizing a Need: Intrinsic Motivations for Treatment

Intrinsic motivation is considered to be important in treatment settings, as it improves engagement within offending behavior programs and, as a consequence, facilitates more long-lasting treatment effects (Ryan & Deci, 2006; Ward et al., 2004). Within the current research, all participants acknowledged an intrinsic motivation for taking MMPSA, driven by the self-identified need for some type of change in their sexual thoughts and behaviors.

That’s why I went on it [the medication] yeh the reason I’m on it is cus like in a real, like in an ideal world I’d want something what lowers my masturbation, to not, I don’t wanna stop wanking I obviously wanna wank like three four times a day, not fucking ten to fifteen times a day yeh in an ideal world (P. 8).

Here, participant 8 discusses the desire to lower his frequency of masturbation, contrasting “real” and “ideal” scenarios. His use of “fucking” demonstrates his exasperation at his current reality of masturbating 10–15 times per day, with his ideal being three to four times per day. In this sense, masturbation has come to dominate participant 8’s life, leading him to recognize that this behavior needs to change. What is interesting though is that his end goal is a reduction in masturbation, rather than a total elimination of masturbation, stressing his desire to not stop masturbating entirely. In this sense, the extract from participant 8 may represent some degree of personal re-orientation in terms of his personal identity, and this may be common among individuals taking MMPSA. This re-orientation, from somebody who is hypersexual (with this being the main aspect of his identity) to being a person with “normal” levels of sexual thoughts and behaviors is an idea that is present in the narratives of other participants. However, for some participants, this “ideal” outcome would still be considered to be excessive:

That’s one thing I think about myself that before I’d masturbate 24/7, three, four, you know, five times a day, and I didn’t really enjoy it, I didn’t want to do it, it was just, that’s to get rid of that frustration and everything else out…because I had this problem, I was thinking about sexual things all the time so I’d feel sexual things all the time so I’d feel aroused all the time (P. 4).

For participant 4, masturbating between three to five times per day is described as “24/7,” suggesting that this was something that consumed every hour of his life. The subjectivity of participants’ perceptions of excessive levels of masturbation is illustrated here, where participant 8’s “ideal” masturbation frequency (three to four times per day) is both classified as excessive by participant four and would far exceed clinical cut-offs for classifying him as hypersexual (Kafka, 1997). These extracts suggest that PSA should not simply be measured by the frequency of sexual thinking or behavior, but should also be considered in relation to an ability to cope with or manage this (including the extent to which PSA interferes with daily life).

Masturbation is commonly used to alleviate stress and frustration (Carvalheira & Leal, 2013), with 15% of men masturbating because they feel like they have to (indicating a compulsive explanation for a substantial proportion of male masturbation; Yule et al., 2017). This trend was also observed in other participant narratives:

It’s annoying when you wake up through the night and you’re so tired but you can’t go to sleep because obviously you got a a an erection and it starts hurting…I'm tired but yet I can’t go back to sleep and I can’t get rid of it, unless I I masturbate so I just do it cus I have to (P. 10).

I kept getting embarrassed nearly every day going to and from work with a like I say erected I was getting so annoyed coz I used to get a semi hard on and everything like that, just really embarrassed and uh that’s where that’s why I asked if I can take is there any medicine or any tablets I can take (P. 1).

This involuntary nature of PSA was frequently discussed within participant narratives. For some, this impacted negatively on other aspects of their lives, as shown within the extract of participant 10 as interfering with his sleep and causing physical pain that can only be relieved through masturbation. This again emphasises the notion that masturbation becomes a necessity, rather than a normal aspect of healthy sexual expression. Participant one discusses his lack of control over sexual arousal in relation to feelings of annoyance and embarrassment. This perceived lack of control was present throughout many of the narratives. Thus, establishing and maintaining a healthy expression of sexuality, and most importantly having control over this, could be a suitable and desirable outcome for men convicted of sexual offences with PSA.

One psychological process that underpins many of these narratives is the concept of learned helplessness (Seligman, 1972), where people tend to give up hope of changing a negative stimulus in their life when they perceive a loss of control over its elimination. In the context of PSA, participants’ learned helplessness is caused by the uncontrollability of their sexual arousal which leads them to believe that they are in some way destined to live a life of unhealthy or harmful sexual patterns without intervention. This is where MMPSA plays an important role in the rehabilitation process, harnessing participants’ intrinsic motivations to gain control (see also Lievesley et al., 2014). They provide a way of managing PSA in a controlled environment and can be used in conjunction with other psychological treatments to encourage a sense of personal agency and facilitate the reduction in a reliance on MMPSA over the longer term (Grubin, 2017). That is, there is a motivational aspect of losing control that leads individuals with PSA to ask “Is there any medicine or any tablets I can take” (p. 1) to attempt to regain control. The decision to engage with MMPSA in the current context (where prescription is voluntary in nature) thus represents an intrinsic motivation by some participants to change their sense of self, rebuild their identity as a person with healthy sexual thoughts (rather than a person dominated by them, similar to participant eight above), and to take control over their long-term behaviors and treatment outcomes. This sense of being able to have control or agency is particularly important in maintaining motivation and engagement in a range of treatment settings, whether this be medical (Bishop & Yardley, 2004), psychological (Adler, 2012), or in relation to [sexual] offending (Göbbels et al., 2012; Maruna, 2001; Maruna & Copes, 2005).

It is not only the effects of physiological sexual arousal and the obvious implications that this has on behavior and everyday functioning that participants felt to be an important motivator of their seeking MMPSA:

I was getting desperate…I knew I had to do something, my obsession with sex, was getting tiring, umm, from the moment I got out of bed in the morning to the moment I went to bed of a night, I was thinking about sex, I couldn’t watch a TV program without sexualizing it, without objectifying the females in it (P. 9).

Similar to how participant 1 reflected on his lack of control over physiological arousal, participant 9 reports a lack of control over his sexual thoughts. His use of the term “obsession with sex” is present in the definition for sexual preoccupation within the now-defunct Structured Assessment of Risk and Need (SARN; Craig & Beech, 2009) for ICSOs, and is highlighted as a dynamic risk factor for recidivism in several meta-analytic studies and empirical reviews (Brankley et al., 2021; Hanson & Yates, 2013; van den Berg et al., 2018). This might suggest that participant 9 possesses good levels of insight that his PSA is out of control, resulting in him “getting desperate” to “do something” to address it. This represents a turning point for him and drives his motivation to engage in treatment to gain control and begin to bring his arousal under control and work towards desistance (Göbbels et al., 2012).

As highlighted here, the majority of participants suggested that their PSA was a constant and enduring issue that impacted upon different aspects of their lives (see also Lievesley et al., 2014). Furthermore, they allude to a perceived lack of control over their sexual thoughts and behaviors, indicating a learned helplessness about their PSA before taking medication. The availability of medication provided these participants with an opportunity to take back some control over their sexual thoughts and move towards their idealized sexual identities. As such, their medication use was in part intrinsically motivated by this desire for personal agency and control.

#### Theme 1.2. Forced Compliance: Extrinsic Motivations for Treatment

For some participants, while there was recognition of an intrinsic motivation, their primary motivation for taking the medication was related to an external factor (e.g., a belief that there were some pressures to comply):

I’m on IPP, I had no choice, I can’t afford not to do these things because everything I try and do for myself shows the board I’ve done this off my own back and I’m trying to change and they can see that I’m trying to change I’m trying to put things in place to try and stop me from reoffending (P. 4).

Here, both participants discuss the impact that their IPP (indeterminate sentence for public protection) sentence had on their decision, with the belief that they had no choice but to consent to MMPSA.^2^ This belief likely stems from the requirement that those serving IPP sentences must ensure that the parole board are “satisfied that it is no longer necessary for the protection of the public for the offender to be confined” (Bradford & Cowell, 2012, p. 1) before they can be released. Participant four emphasises that everything he does “shows the board” that he is trying to change and reduce his risk, with a recognition that he will not be released until achieving this.

The extracts echo a sense of desperation, born out of a need to progress towards release. This is not uncommon among IPP prisoners, with many several years beyond their tariff (minimum sentence term) date. This leaves them “stuck” in the system, doing what they can to try to demonstrate a reduction in risk without the hope or security of a definitive release date (Annison, 2018). For the participants here, consenting to take MMPSA presented an opportunity to move towards release, by doing something “off my own back” (due to the voluntary nature of the treatment). This is an important finding, as having agency over one’s life and hope for the future are key aspects of the desistance process for ICSOs (Gӧbbels et al., 2012). However, when hope and control feels unattainable (either in relation to one’s sexual urges and behaviors, or the length of a prison sentence), engaging with the medication presents a plausible way of overcoming this, although potentially only in the short term (see below). This view was not just restricted to those serving IPP sentences, but also more generally for those serving life sentences with the possibility for parole:

Yes I have a lot of sexual thoughts but there is no harm in masturbating yeah, like as much as I do. Yes it’s out of the ordinary, yes it’s a bit extreme but it’s not harming no one… the only reason I wanted to go on it is for one, to look good on the parole, cus I know it does yeah, I'm not stupid, some people say no it might not or it won’t, but I know it does…so this is my compromise for that (P. 8).

While participant 8 acknowledges his high level of sexual thoughts and masturbation to be “extreme”, at the same time, he is defending this in arguing that “it’s not harming no one”, fuelling his belief that the medication is not needed. For him, the medication is the result of a “compromise” to show the parole board he is taking on board recommendations and actively trying to reduce his sexual arousal. In doing so, he believes that this would look good and increase his chances of release. This perspective that taking the medication would “look good at the parole board” (P. 2) or ensure they “get out quicker” (P. 7) was held by a number of participants. Research with offender managers in prison has found that they hold a sceptical view of the medication for this very reason (Elliott et al., 2018).^3^ Despite this, the voluntary nature of taking MMPSA may suggest to some individuals taking MMPSA that members of the parole board could view them as more motivated and engaged in the process of addressing their PSA, and therefore see them as a lower risk. This perceived external pressure to take with MMPSA also relates to views regarding the use of the medication post release from prison:

When I get released erm, like I said I've got some licence left to do so I’ll still take the medication cus it’s on my licence conditions but once I’m off licence I will carry on taking them for a while and just gradually take myself off them (P. 7).

Despite MMPSA being voluntary in the UK, and HMPPS guidance stating that it should not feature as a licence condition or requirement for release, a number of participants held this belief. The effect of this is that they believe that taking MMPSA is something that they must continue with for the duration of their licence period. Collectively, these extracts demonstrate how compliance with MMPSA can sometimes feel forced. While this is indeed the case in countries where the medication is mandatory, this is not the case for our participants, demonstrating a lack of understanding regarding the voluntary nature of the treatment. This highlights the need for accurate information to be provided to such individuals who may feel coerced into taking MMPSA, which is a fundamental aspect of the ethical prescribing of MMPSA for ICSOs (Turner et al., 2019). For participants 7 and 4, it is clear that once the perceived external pressure for taking the medication is removed, they no longer intend to take it, with both participants discussing how they will navigate the process of ceasing MMPSA. Most concerningly, participant 4 describes how he will “see how it goes” when stopping medication, referring to this as a “test.”

The reduction in legal restraints and external pressures leading to a decline in treatment motivation has been indicated elsewhere in the literature on anti-androgen treatment in individuals who have committed sexual offences (Voß et al., 2016; Wolba et al., 2023). This is further supported in the wider forensic literature; when individuals are extrinsically motivated or feel coerced to comply with treatment programs, extended engagement with interventions is poor (Day et al., 2004; Sturgess et al., 2016). This highlights the importance of identifying potential sources of intrinsic motivation and establishing viable future identities before starting MMPSA. Through a process of contemplation (Prochaska & DiClemente, 1983) therapists can work with service users in order to make sure that they are ready to change and engage fully in treatment (Barrett et al., 2003), which has been identified as an important predictor of treatment success among ICSOs (Göbbels et al., 2012; Sowden et al., 2017). In doing so, intrinsic motivations may act as a buffer to negative experiences of MMPSA treatment, enabling service users to continue to have the required motivation to adhere to the treatment program when side effects are severe or when extrinsic pressures to comply are removed.

### Theme 2: Medication as a Risk Management Strategy

#### Theme 2.1: “Too Risky” Without Medication

Throughout the narratives there was an awareness of and recognition that participants are unable to manage their sexual thoughts and behaviors on their own and are, in essence, considered to be “too risky” without medication.

I know that on my own, I’m not strong enough to deal with this, I’ve been doing this since I was 13 years old and I’m not strong enough without it, I don’t trust myself without it (P. 9).

Here, both participants discuss their need for the medication. Participant 9 suggests that, without medication, he cannot “deal with this” (his PSA), and alludes to the view that this increases his risk of reoffending. Participant 1 is much more direct in discussing his views regarding this. For him, being released into a much less controlled environment where there are children presents an increased risk, and medication is viewed as a method for managing this. In this sense, the medication is seen as a protective barrier to reoffending.

Several participants had previously received SSRIs before acknowledging that these were ineffective or insufficient for addressing their PSA:

Yeah I before I was on fluoxetine but that wasn’t doing nothing for me so…he decided to then put me on that and this new one a stronger one but I didn’t see the point in that one I knew it wasn’t doing anything so now I’m just on more of the stronger one which is working (P. 7).

Here, there is the suggestion that SSRI medication did “nothing” (i.e., had no impact on their PSA) or was insufficient which led to adjustments being made to their medication. For participant 7, this involved initially progressing to a combination of SSRI and CPA, before an increased dose of CPA alone, whereas for participant 3, this was a move straight to CPA. This lack of perceived effect of past SSRI treatment may be real, but may also be related to the subjective nature of experiences of PSA, as indicated previously. SSRI treatment has been found to have positive effects on indices of PSA (Lievesley et al., 2014; Winder et al., 2014, 2018). For the participants here, though, it may be that such improvements did not match their personal targets. There are also parallels in this progression from one medication class to another with the literature around medication tolerance, with the body becoming used to a particular dose before something more potent is required to have therapeutic effects. These narratives highlight the complex nature of MMPSA treatment journeys. That is, these participants all started on SSRIs and ended on anti-androgens, but their individual journeys between those two points were distinctly different. It is therefore clear that a “one size fits all” approach to the medication is not appropriate, highlighting the important role of professional clinical judgement and service user collaboration when prescribing MMPSA.

This concept of feeling “too risky” without medication is not without potentially negative consequences. While some participants felt that the medication would be viewed positively by parole boards, others felt the opposite, in that needing the medication to reduce their risk could be viewed negatively.

All I think of is what [name] said about it the other day, that the parole board will see it as it being dangerous to release someone like me, someone on meds, how high my sex thing is (P. 5).

These participants articulated the belief that having a need for medication increases their perceived risk of reoffending. For participant 5, having high levels of PSA, and being on medication for this, is viewed as his core defining feature in his use of “someone like me…”, and that until this changes, he will be viewed by the parole board as too dangerous for release. Similarly, participant 1 discusses the perceived increased risk due to his lack of control and thus, need for medication in that he would become “too risky” if he chose to stop taking them, echoing his earlier comments. These ideas speak to these individuals living out a condemnation script (Maruna, 2001) where they see themselves as being doomed to a life of sexual crime as a result of their PSA. A lack of perceived ability to change may also hinder the rehabilitation process itself. That is, seeing a non-offending identity as being unviable prevents an individual from orienting themselves towards a reformed state of being. This ‘new me’ is said to be an important aspect of long-term desistance from sexual offending as it is an intrinsic motivation for change (Gӧbbels et al., 2012; Ryan & Deci, 2006), thus increasing the likelihood of deeper and more lasting engagement in treatment interventions (Adler, 2012; Bishop & Yardley, 2004).

The notion that some ICSOs believe that MMPSA could make them appear more risky might prevent individuals who recognize PSA within themselves from disclosing this. This could subsequently act as a barrier to treatment if the perceived costs (e.g., hindering release) outweigh the potential benefits (e.g., reduced arousal and sexual risk (Burrowes & Needs, 2009). Further research is required to empirically understand whether professional risk assessments of ICSOs are actually affected by them taking MMPSA, with the results of these studies being used to alleviate any such fears, or provide information to ICSOs about how perceptions could change as a result of their treatment choices.

#### Theme 2.2: “One a Day Keeps the Prison Away”

Having recognized that their PSA may be a risk factor for their offending, or may be viewed as such by others (e.g., offender managers, psychologists, and parole boards), participants suggested that this risk is directly managed by the medication:

I said I want something to stop me offending. The tablets will stop me offending…it’s like a cure (P. 5)

I’m thinking as, I’m thinking now, one a day keeps the doctor away or keeps the prison away, ahh that’s good one a day keeps the prison away yeah (P. 2)

As depicted in the extracts above, participants held the view that the medication would simply stop them from offending and would preventing them from returning to a life of offending. In this sense, the medication is viewed as something that eliminates all potential risk, thus removing personal agency from the rehabilitation process, which subsequently has the potential to hinder the process of desistance among ICSOs (Gӧbbels et al., 2012). These beliefs could also create an overreliance on the medication, as highlighted in participant nine’s use of “it’s like a cure.” This mindset may prevent ICSOs who are prescribed anti-androgens reflecting on broader risk factors that led to the offending, which is important when a recognition of these factors is associated with reduced reoffending risk (Bushman & Van Beek, 2003).

This mindset is also potentially problematic as, if participants feel they had no control over their offending due to PSA, and the risk of this is now eliminated by the medication; it may lead to a less pro-active approach to their desistance due to the belief that they are now “cured.” However, some participants have begun to move past this viewpoint and adopt different strategies:

I thought that the tablet I was taking every day was supposed to stop these thoughts erm I didn’t realise at the time that it works in conjunction with me, it’s got to come from me, it’s got to be my desire to change, these tablets will help me but I thought that taking these tablets and that would be it, I wouldn’t have to do anything…but I did erm it’s not a magic pill, I’ve had to change the way I think (P. 6).

Here participant 6 alludes to the belief that he previously held that the medication would stop the thoughts, with this counteracted by the recognition that “it’s not a magic pill.” Instead, he reflects on the need to be motivated and want to change, and take the necessary steps to change alongside taking the medication. A method of achieving this, for some, was through engagement in offending behavior programs while taking the medication:

The meds help me think clearly, I’m not aroused all the time and I’m not thinking about sex all the time, I can sit in like programs and focus on what’s going on and been learning strategies and everything like that…I’ve got con, I’ve finally got control over myself… (P. 9).

Both participants 9 and 3 discuss the combined effect of medication and psychological interventions. For participant nine, this is about gaining control over his sexual thoughts and behaviors, with the medication providing the headspace that is necessary to focus when engaging with offending behavior programs and develop appropriate strategies to manage sexual thoughts and arousal (see also Lievesley et al., 2014). The increased effectiveness of combined treatment is widely accepted (e.g., Guay, 2009; Saleh et al., 2010; Thibaut et al., 2020), with MMPSA prescribing guidelines also recommending this approach (Grubin, 2017). Developing the necessary strategies to manage sexual thoughts in such programs, as participant 8 articulates, is important when considering long-term plans for desistance, particularly as medication may not work or be taken indefinitely (Gordon & Grubin, 2004). This cessation of medication may result in the return of PSA, as demonstrated in individuals taking MMPSA who choose to stop taking MMPSA (Lievesley, 2019). As suggested previously, the headspace that MMPSA creates thus allows ICSOs to work on acquiring the necessary cognitive and behavioral skills to manage sexual arousal in less controlled environments upon release.

#### Theme 2.3: A Small Price to Pay

The negative aspects of the medication were acknowledged and discussed by participants; however, the dominant discourse suggested that these were “worth it”, indicating that consent and compliance to MMPSA is the result of a rational choice (Becker, 1978) after considering the apparent benefits of reducing (or appearing to reduce) PSA and perceptions of sexual risk:

I was a bit um dubious because you hear these stories of like erm taking the medication an men who grow breasts an you know all like this (P. 4)

I wasn’t really sure about it because I’d heard from other blokes that umm, how you start taking this and it kills you completely like chemical castration well I was, I was unsure about that, I really didn’t want that...but I asked Dr [name] and he was able to put my mind at ease straight away…once you stop taking it, after a certain length of time everything’s back to normal again so it’s fine (P. 9)

Here, both participants discuss the impact of hearing others talk about the effects of the medication instilling a sense of uncertainty regarding whether they wanted to take the medication (see also Elliott et al., 2018; Lievesley et al., 2014). It is important to note that some of these rumours do represent accurate information, with documented side effects of anti-androgens including gynecomastia (breast growth) and an inability to gain an erection (Nguyen et al., 2015). It is also true that anti-androgens are used in some countries to achieve a state of chemical castration (Douglas et al., 2015). While these effects are reversible (CPA is fully removed from the body within approximately 10 days of discontinuation; Nnane, 2019), having accurate information readily available would reduce anxiety and uncertainty regarding the longevity of such effects.

While participant 9’s concerns were eased through discussions with the prescribing psychiatrist, these issues remain worrying for those who find accurate information more difficult to come by:

I’m gunna always take these until they say oh there’s no point or whatever. I don’t particularly want to cus I think, you know I’m young and with the side effects sort of thing, I don’t want anything to go wrong with my sperm or whatever, you know obviously I want a family and err I don’t wanna risk anything but, if it’s gunna help me get out, then I’ll take it and I’ll keep taking it (P. 3)

Within both extracts above, the participants acknowledged how they do not want to take the medication because of potentially serious side effects (e.g., infertility), but they consider this within the context of MMPSA improving their chance of release. In this sense, the benefits of the medication (helping towards their release) far outweigh the costs (potential side effects) resulting in consent to taking the medication regardless of any potential adverse effects. This resonates with the discussion in the previous theme of forced compliance, suggesting that perceived external coercion is a typical motivation for engaging with MMPSA (Garcia & Thibaut, 2011).

Some participants reported a number of adverse effects of anti-androgen MMPSA, such as weight gain (P. 8), tiredness (P. 6), and an inability to maintain an erection (P. 2). However, the concerns and side effects were all considered “a small price to pay” (P. 9) in return for the perceived positives of the medication. Importantly, however, while this may be the case presently, this perspective may change longer term if participants were to begin experiencing some of the more severe side effects such as gynecomastia or long-term sexual dysfunction. As such, individuals taking MMPSA should be carefully monitored, as advised within the prescribing guidelines and wider recommendations related to the use of MMPSA (Grubin, 2018), to maintain their motivation and compliance with treatment.

### Theme 3: Discovering a “New Me”

#### Theme 3.1: Adjusting to New Sexual Norms

Participants discussed a number of ways in which their sexual thoughts and behaviors had changed as a result of the medication:

Well it’s just reducing my sexual thoughts. I haven’t had one for god knows how long now (P. 5)

Here, the participants reflect on the reduced frequency of sexual thoughts as a result of the medication. Both participants convey that their sexual thoughts are now “gone”—even appropriate ones. Participant 7 articulates the “risk” he perceives in having some sexual thoughts, which could lead to more sexual thoughts and subsequently to problematic behaviors (for a discussion of such escalation, see Lievesley, 2019). As a result, he is “glad” about no longer having any sexual thoughts, a perspective that was generally shared among the participant group.

While the reasons for being pleased about these changes are not explicitly articulated in the extracts, they may be linked to changes in levels of PSA being associated with moves towards the idealised identities developed in the contemplation and preparation stages of change (Prochaska & DiClemente, 1983), with observable changes in levels of PSA helping this “new me” identity to become crystallised. This subsequently serves to further increase intrinsic motivations to maintain engagement and compliance with treatment and continue their journeys towards desistance (Gӧbbels et al., 2012). While the above extracts highlight changes in sexual thoughts, participants also identified changes in their sexual behaviors:

…since the medication I haven’t [had sexual thoughts or masturbated] and that’s it. Nil, nil, nil all the way down, no masturbating, no erections, no fantasies (P. 2)

Both participants use repetition to emphasise their lack of sexual arousal and behaviors. While a reduction in arousal is consistent with the narratives of those receiving SSRI medication (Lievesley et al., 2014), those taking anti-androgens participants portray these changes to be more absolute in nature. Some of the participants reflected on these changes:

I’ve got so much free time now, so many free hours from when yeh well when I used to masturbate 5 erm 5, 6 times a day and now none…so yeh I think I’m still adjusting to that (P. 6).

Participants reflected on the uncertainty caused by these changes, particularly for participant one in descriptions of his new patterns of behavior as being “weird.” As participant 6 articulates, this is not a simple process of accepting the change, but is instead a process of ongoing adjustment. This process caused apprehension among some participants:

I obviously knew what it was meant to do erm reducing the thoughts and all that but I didn’t expect to not have any, you know it just cut it out, and I’m glad I’m happy now you know but erm at first it er it erm felt like I’d lost part of me because I was so used to always having them (P. 4).

For participant 4, his expectations of change differed from reality. The perceived drastic removal of sexual thoughts and behaviors initially left him feeling as though he had “lost” a part of himself. This was a feeling shared by other participants, particularly when the sexual thoughts and behaviors had previously acted as emotional coping mechanisms (see Carvalheira & Leal, 2013; Lievesley et al., 2014; Yule et al., 2017). Not only did this loss mean that participants had to replace a physical need, but also an emotional coping strategy (Bancroft & Vukadinovic, 2004; Brewer & Tidy, 2019; Cortoni & Marshall, 2001; Hughes, 2010; Walton et al., 2017), leading to the need to fill the void with new hobbies and activities. This again highlights the need to fully inform service users about the likely physical and emotional effects that they will experience, and to preparing them for emotional turbulence during the change process (see Berking et al., 2011; Brown & Bloom, 2009; Liebling, 2012).

As previously discussed, participants expressed a desire to reduce their sexual thoughts and behaviors but did not want to lose these completely. This desire is consistent with the aims of MMPSA, for which guidance is explicit in that the medication should reduce PSA while maintaining the ability to express a healthy sexual identity (Grubin, 2018). While previous research has found that SSRI treatment still allows for some sexual thinking (and therefore the reinforcement of appropriate fantasies and thoughts; see Lievesley et al., 2014), anti-androgens appear to simply eliminate all sexual capacity which left some participants with difficulties in adjusting to their new sexual norms from an emotional and identity perspective. This observation should be considered within the context of the various trade-offs between the costs of the medication (i.e., the loss of sexual arousal) and the benefits that it brings (i.e., the chance of a new start, new hobbies, and reduced risk), but highlights a need for careful planning and monitoring to maintain compliance and service user involvement with the treatment process.

#### Theme 3.2: An Awakening

Participants discussed the medication, or rather the changes they experienced from the medication, as giving them a ‘second chance at life’:

It’s just given me a, a second life, I would put it, it’s given me a better life than the one I was leading before because all I was into was sex, sex, sex, sex, sex mad (P. 3)

Participant 2’s use of being “in the dark” is used to represent a lack of understanding, echoing participant six within the previous subordinate theme who did not realising anything is wrong until taking MMPSA. This “darkness” represents them being in a pre-contemplative stage of change prior to engaging with the medication (Prochaska & DiClemente, 1983). Both participants’ discussion of a second (chance at) life, suggests far more than simply a chance to change. Instead of being consumed by sex, participants could reflect on the wider impact of the medication and what it allows them to achieve. As participant two articulates, this is the ability to become somebody new, to develop a new identity, and to “think and do things I couldn’t do before.” This is something also discussed by other participants:

Being on these tablets have helped a heck of a lot…I can watch my programs that I couldn’t watch before um (P. 1)

Here, both participants discuss their ability to now engage in relatively mundane activities that would have been problematic due to their sexual arousal. For these participants, the void left by the removal of PSA has been filled by prosocial activities. The development of such activities is desirable from a rehabilitation perspective and link to the primary human goods depicted in the Good Lives Model (Ward & Brown, 2004; Ward et al., 2007). Previously, participants would meet this need in destructive or problem-enhancing ways (e.g., engaging in deviant fantasies and masturbating to these). However, the reduction in PSA experienced as a function of the anti-androgen medication has allowed (or, as identified in the previous subordinate theme, has required) them to develop more positive alternatives. This process facilitates the desistance process for ICSOs (Gӧbbels et al., 2012) by encouraging a range of prosocial activities to be developed. By developing these skills in custody, ICSOs stand a better chance of normalising these new routines more quickly upon their release (e.g., Caulfield et al., 2016; Nugent & McNeill, 2017).

Once I started taking the meds it all became clear…I realised now what I had put hundreds, excuse me, hundreds of women through, alright, I hadn’t physically attacked them, no, I had done worse, it’s the surreptitious looking, looking where I’m not supposed to be looking, looking without being given permission to look, this is the realization, you know, the turning on of the light, I suddenly realised and now I’ve got another chance to make amends and to be different and not just be known for being the person who is always looking, always waiting to look, waiting to get an eyeful, instead people might start to see me as something else, something better and people have already started to notice, wing staff and [psychologist] have already said they’ve noticed the change in me (P. 7).

Having time to think and reflect without the distraction of his arousal allowed participant 7 to acknowledge the extent and impact of his offending, with the recognition that his offending behaviors defined him as “the person who is always looking.” However, the medication provides an opportunity for him to change that, and to be noticed for something different. This allowed him to positively re-define himself and re-story his life going forward and echoes the sentiments of participants 2 and 3 discussing their second chance at life. In this sense, participants are using their new identity to “signal” the early process of desistance (Maruna, 2012) and provide an identity to live up to during this process of change. For participant 7, having recognition from others of this change (“wing staff and [psychologist] have already said they’ve noticed the change in me”) will encourage him to behave in ways consistent with the change he has presented, reaffirming his new reformed identity (Maruna et al., 2004).

I don’t think my sex drive is anywhere dangerous now cus I know what I’m like, you know I know I’m in control of myself now…I’m not the person I used to be, that was the old me, I’m you know I’m different now…I don’t think them things or do them things no more since the tablets, I’m a new me now (P. 8).

Participant 8 explicitly re-defines himself using “new me.” He acknowledges that at one point his sexual arousal was dangerous and out of control, while emphasising that he is “different now.” In this sense, by construing “the old me” as the stigmatized part (someone with PSA convicted of a sexual offence), he is in a sense “knifing off” (Maruna & Roy, 2007) these past elements of himself and leaving these behind. This rids himself of stigma, allowing him to move forward with a new identity on his desistance journey (Gӧbbels et al., 2012).

### Conclusions

In this paper, we have presented the first in-depth phenomenological look at the experiences of men voluntarily taking anti-androgens in a prison environment, to treat PSA after committing a sexual offence. This is a significant investigation, as the current knowledge on the use of medication for treating PSA is limited to quantitative studies of changes in indices of arousal and risk (for reviews of this literature, see Darjee & Quinn, 2020; Grubin, 2018; Turner et al., 2018). What was missing until the current study was an examination of the lived experiences of men engaging with this treatment to understand how they felt about receiving the medication, the effects it has on their everyday functioning, and how clinicians might best encourage both engagement and compliance with the medication. This is the original contribution of the work presented here.

### Summary of Findings

While participants generally had positive experiences in terms of reductions in PSA, for some, this “journey” (P. 6) was sometimes fraught with apprehension and uncertainty. The findings highlight the differing motivations for the medication, with all participants acknowledging an intrinsic motivation to change in their sexual arousal, whether that be thoughts or behaviors. Importantly though, these narratives also highlighted the subjective nature of what may be viewed as problematic by each individual. As a result, the intended gains of the treatment should also be individualized.

This observation has the potential to significantly change how PSA is viewed in such treatment contexts and highlights the importance of the methods used here to understand participant experiences. That is, if we rely on clinical cut-offs and statistical differences in indices of PSA over time to determine treatment success, we risk missing the nuances of how treatment is perceived by service users. By way of an example, participant eight reported how his “ideal” level of masturbation was three-to-four times per day, which far exceeds clinical thresholds for hypersexuality (Kafka, 1997). As such, his treatment could be classified as a failure due to his absolute rates of masturbation, even if he personally feels more at peace with his sexual behavior. In contrast, participant four identified a much lower ideal rate of masturbation. He could, therefore, demonstrate the same clinical changes as participant 8, but by virtue of his personal feelings about his arousal my lose his motivation to continue engaging with treatment. As such, a shift in how we view PSA—from a construct that can be ascribed a threshold level of sexual thinking or activity, to one that also considers the effects of arousal on everyday functioning—is supported by these narratives.

Despite this intrinsic motivation, for many participants, the primary motivation was related to an external factor—largely linked to their indeterminate sentence and wanting to demonstrate a reduction in risk in order to progress towards release. Typical sentiments included that consenting to the medication will help them to “look good at the parole” (P. 2) or “get out quicker” (P. 7). In contrast, others report a concern that a need for medication makes them look “too risky” to be released. In either of these cases, there may be implications for treatment engagement, as those who are intrinsically motivated are more likely to maintain a such engagement over the longer term (Sturgess et al., 2016), particularly when the potentially severe side effects of taking anti-androgens are considered (Nguyen et al., 2015). These narratives point towards a fundamental misunderstanding of the voluntary nature of the medication and as such there are some significant implications for the ways in which clinicians communicate with potential service users. Ethical prescribing requires service users to make fully informed decisions about their treatment. This means that service users need to be in complete knowledge that receiving anti-androgen medication plays no formal role in decisions about their risk (and/or release). In enacting this recommendation, clinicians can remove extrinsic motivations to comply with treatment and encourage engagement via intrinsic goals, with this being more likely to lead to successful treatment outcomes (Göbbels et al., 2012; Ryan & Deci, 2006). This is also likely to have longer-term effects in encouraging the maintenance of such voluntary treatment once external pressures (e.g., needing to demonstrate a reduced level of risk, or parole conditions) are removed (see also Day et al., 2004). This is important as the current study, as well as other recent research, highlights that some individuals consider stopping treatment after parole, and most do so without discussion with their clinicians (Sauter et al., 2022; Wolba et al., 2023).

Regardless of motivation, participants’ primary concerns appear most related to a lack of control—either over their sexual arousal (intrinsic) or their sentence and ability to demonstrate a reduction in risk (extrinsic). In both scenarios, the medication presents a potentially viable way of gaining control over these issues. For some, the medication was viewed as a “cure” for their PSA, with them suggesting that it may have even prevented offending before it occurred, had it been available. While MMPSA is not used in a preventative way within the UK, and is only used with ICSOs, this is an approach used in other contexts internationally, such as in the prevention of child sexual abuse (Amelung et al., 2012; Konrad et al., 2018). As preventative efforts are continually evolving, the use of MMPSA outside of the criminal justice system may be considered for those individuals who recognize a treatment need and are seeking support to prevent them from offending, or if healthcare providers feel that this is appropriate.

This research highlights that while initial decisions to use anti-androgens may be driven by a perceived last chance to regain control, anti-androgens do reduce PSA, supporting past quantitative evaluations with an additional phenomenological layer (see Darjee & Quinn, 2020; Grubin, 2018; Turner et al., 2018; Winder et al., 2014, 2018). While these stark reductions in arousal were not always desired to begin with, participants adjust to their “new sexual norms” and begin to construct a “new me” identity. This is important as being able to regulate emotional states and engage in prosocial recreational activities predict desistance among ICSOs (Gillespie et al., 2012). By freeing participants from the constraints of their PSA, the medication not only helped but required them to develop new coping strategies in relation to emotional regulation and find other activities to occupy their time—issues that people with high levels of hypersexuality typically struggle with (Walton et al., 2017). However, this needs to be balanced with the observation that some participants saw the medication as a “cure” for their offending, which might limit their motivations to engage meaningfully with psychological interventions.

### Clinical Implications

An implication of the observations made in this analysis relates to the importance of a more holistic approach to treatment, with service users being made aware of the need to engage in both pharmaceutical and psychological support. In the long run, the effective use of such a combined approach should have a positive effect on the ease and speed at which these individuals will be able to re-enter the community at the end of their prison sentence (Gӧbbels et al., 2012; Grubin, 2018; Guay, 2009; Saleh et al., 2010; Thibaut et al., 2020). Length of treatment should however be considered alongside the updated WFSBP guidelines and ethical considerations surrounding side-effects of anti-androgens (Thibaut et al., 2020). Studies have demonstrated that pharmacological treatment can be stopped without an immediate association with increased risk of sexual recidivism (Sauter et al., 2018; Voß et al., 2016); however, navigating the journey of discontinuing medication is a complex decision, which can lead individuals to stay on their medication for possibly longer than necessary periods (Sauter et al., 2021, 2022).

From a process perspective, the importance of fully informed consent has been brought to the fore in our analysis. This is of particular importance within the context of a perception of forced compliance, with this research highlighting how clear, accurate and consistent communication is needed to ensure service users are secure in their understanding of the voluntary nature of engaging with the treatment pathway. At present, this is not the case, with service users clearly perceiving some external pressure and it is not currently known how accurate this may be (discussed below). A key issue that came from our sample, particularly among men serving indeterminate sentences, was a perceived issue of forced compliance There were also significant challenges faced by participants related to anti-androgen side effects that must be taken seriously. With many of the current participants reporting a desire to discontinue medication use in the future, individuals must (1) engage with anti-androgens while knowing the potential side effect profile, and (2) be sufficiently prepared and supported to come off the medication gradually and under supervision to allow changes to sexual arousal to be carefully monitored. It is currently unknown how compliance with MMPSA looks within community settings. This further supports the argument for MMPSA to be combined with psychological treatment (Grubin, 2018; Guay, 2009; Saleh et al., 2010; Thibaut et al., 2020) to ensure individuals are developing the necessary skills to manage their sexual arousal independently if it was to return after treatment discontinuation.

This complex decision may in part be understood by one of the overarching observations within this study that the MMPSA treatment pathway is not a straightforward one. Participants frequently highlighted the process of regular changes between medication type and dosage. This was also discussed by those taking SSRIs within the same treatment context (Lievesley et al., 2014). However, the true extent of this became apparent within the current analysis, with several participants beginning on SSRIs and eventually progressing to anti-androgens. Each individual journey between these points was very different, which accentuates the view that a “one size fits all” approach to the medication does not work (Thomas & Daffern, 2014). Although practice guidelines exist, these are typically geared around the initiation of treatment, with SSRIs used as a first-line option for those with more psychologically driven PSA, and anti-androgens suggested as being appropriate for those with hypersexual behavior (Grubin, 2017). The CostLow (Change or Stop Testosterone-Lowering Medication) scale is one example of a possible structure for clinicians to use in the decision to change or discontinue pharmacological treatment (Briken et al., 2019; Wolba et al., 2023). However, further exploration of the nuanced pathways within the MMPSA treatment pathway, including how service users begin, adapt, and switch between different drug classes, is therefore a vital next step in developing a full understanding of the MMPSA treatment pathway.

### Limitations and Future Directions

What might be considered the most obvious limitation of this work is the focus on the lived experience of ICSOs, rather than a quantitative investigation of the effectiveness of MMPSA. More quantitatively oriented studies as to the effectiveness of anti-androgens on indices of risk, for example, have been presented elsewhere (see Amelung et al., 2012; Briken et al., 2001; Turner et al., 2013; Winder et al., 2014, 2018). The current study aimed to supplement this quantitative work with a greater richness and depth of understanding to contextualise these quantitative evaluations.

Methodologically, the use of just one prison site may limit the generalizability of the findings presented here. Although generalizability to whole populations is never the aim when conducting phenomenological work, the site chosen is known for its therapeutic excellence and as such it may be that those taking anti-androgens at other (perhaps less therapeutically oriented) sites would report different experiences. Generalizability to the specific site can be said to be high, though, as the sample here reflects all individuals receiving anti-androgens as a form of MMPSA in this prison at the time of data collection. Future work might look to investigate this context effect by examining the role of the social environment (e.g., therapeutic climates) of prisons on the experience and outcomes associated with anti-androgen medication.

As highlighted previously, the analysis presented in this paper suggests that ISCOs’ journeys through the process of being prescribed anti-androgens as a form of MMPSA, and then continuing with these over an extended period of time, does not follow a consistent pattern. This hints towards the existence of different pathways through MMPSA, with the identification of these being an important next step in the development of ethical and responsive clinical practice guidance. A comprehensive analysis of cohorts receiving MMPSA would likely uncover different subgroups, each with specific therapeutic pathways.

A key issue that came from our sample, particularly among men serving indeterminate sentences, was a perceived issue of forced compliance. Although all potential service users are informed that engagement with the MMPSA treatment pathway has no effect on sentence planning (i.e., that MMPSA is not considered in either a positive or negative manner with regard to risk at parole hearings), there is no empirical data on this topic. As such, although the use of medication is not designed to indicate an increase or decrease in risk, it is unknown whether risk assessors actually adjust their evaluations of service users as a function of prescription status. As such, there are questions to be asked about whether consent to engage with MMPSA is currently fully informed, as there is doubt over the veracity of some of the claims made about the sentencing-related effects of taking MMPSA. With this in mind, it is important that risk assessors be studied in relation to their attitudes towards MMPSA, people taking medication to address their sexual arousal, and how prescription status affects risk-based judgments. While the current analysis provides a significant contribution to our understanding of MMPSA, there is more work still to do to understand and enhance our prescribing practices.

## Footnotes

1 Although some elements of mild sexual preoccupation might have been captured in older Sex Offender Treatment Programmes (SOTPs), this was only as a by-product of working on other treatment targets. It is not targeted within the current suite of programs. The former Healthy Sexual Functioning Programme did address this to some extent; however, this also needed to be accompanied by offense-specific sexual interests to be eligible as it did not target sexual preoccupation alone. This has since been updated to the Healthy Sex Programme v2 which does not target sexual preoccupation (Elliott & Martin, 2023).

2 Following a review, IPP sentences were abolished in 2012; however, individuals sentenced to these prior to this date were required to continue to serve these sentences (Bradford & Cowell, 2012).

3 Offender managers in prison are specialist staff, sometimes a probation officer, who manage an individual's sentence during their time in custody.

## Declarations

### Availability of Data and Material

Not applicable—due to the sensitivity of the data we are unable to share transcripts from this research.

## References

[CR1] Adler JM (2012). Living into the story: Agency and coherence in a longitudinal study of narrative identity development and mental health over the course of psychotherapy. Journal of Personality and Social Psychology.

[CR2] Amelung T, Kuhle LF, Konrad A, Pauls A, Beier KM (2012). Androgen deprivation therapy of self-identifying, help-seeking pedophiles in the Dunkelfeld. International Journal of Law and Psychiatry.

[CR3] Annison H (2018). Tracing the Gordian knot: Indeterminate-sentenced prisoners and the pathologies of English penal politics. Political Quarterly.

[CR4] Bancroft J, Vukadinovic Z (2004). Sexual addiction, sexual compulsivity, sexual impulse disorder or what? Towards a theoretical model. Journal of Sex Research.

[CR5] Barrett M, Wilson RJ, Long C (2003). Measuring motivation to change in sexual offenders from institutional intake to community treatment. Sexual Abuse.

[CR6] Becker GS (1978). The economic approach to human behavior.

[CR7] Berking M, Margraf M, Ebert D, Wupperman P, Hofmann SG, Junghanns K (2011). Deficits in emotion-regulation skills predict alcohol use during and after cognitive–behavioral therapy for alcohol dependence. Journal of Consulting and Clinical Psychology.

[CR8] Bishop FL, Yardley L (2004). Constructing agency in treatment decisions: Negotiating responsibility in cancer. Health: An Interdisciplinary Journal for the Social Study of Health Illness and Medicine.

[CR9] Boons L, Jeandarme I, Vervaeke G (2021). Androgen deprivation therapy in pedophilic disorder: Exploring the physical, psychological, and sexual effects from a patient’s perspective. Journal of Sexual Medicine.

[CR10] Bradford S, Cowell P (2012). The decision-making process at parole reviews (indeterminate imprisonment for public protection sentences).

[CR11] Bradford JMW, Kaye NS (1999). Pharmacological treatment of sexual offenders. American Academy of Psychiatry and Law Newsletter.

[CR12] Bradford JM, Pawlak A (1993). Effects of cyproterone acetate on sexual arousal patterns of pedophiles. Archives of Sexual Behavior.

[CR13] Brankley AE, Babchishin KM, Hanson RK (2021). STABLE-2007 demonstrates predictive and incremental validity in assessing risk-relevant propensities for sexual offending: A meta-analysis. Sexual Abuse.

[CR14] Braun V, Clarke V (2006). Using thematic analysis in psychology. Qualitative Research in Psychology.

[CR15] Braun V, Clarke V (2021). One size fits all? What counts as quality practice in (reflexive) thematic analysis. Qualitative Research in Psychology.

[CR16] Brewer G, Tidy P (2019). Sex addiction: Therapist perspectives. Sexual and Relationship Therapy.

[CR17] Briken P, Kafka MP (2007). Pharmacological treatments for paraphilic patients and sexual offenders. Current Opinion in Psychiatry.

[CR18] Briken P, Nika E, Berner W (2001). Treatment of paraphilia with luteinizing hormone-releasing hormone agonists. Journal of Sex and Marital Therapy.

[CR19] Briken P, Turner D, Thibaut F, Bradford J, Cosyns P, Tozdan S (2019). Validation of the Change or Stop Testosterone-Lowering Medication (COSTLow) scale using the Delphi method among clinical experts. Journal of Sex & Marital Therapy.

[CR20] Brown M, Bloom B (2009). Reentry and renegotiating motherhood: Maternal identity and success on parole. Crime & Delinquency.

[CR21] Burrowes N, Needs A (2009). Time to contemplate change? A framework for assessing readiness to change with offenders. Aggression and Violent Behavior.

[CR22] Bushman J, Van Beek D (2003). A clinical model for the treatment of personality disordered sexual offenders: An example of theory knitting. Sexual Abuse.

[CR23] Carvalheira A, Leal I (2013). Masturbation among women: Associated factors and sexual response in a Portuguese community sample. Journal of Sex & Marital Therapy.

[CR24] Caulfield LS, Wilkinson DJ, Wilson D (2016). Exploring alternative terrain in the rehabilitation and treatment of offenders: Findings from a prison-based music project. Journal of Offender Rehabilitation.

[CR25] Craig LA, Beech A (2009). Best practice in conducting actuarial risk assessments with adult sexual offenders. Journal of Sexual Aggression.

[CR26] Colstrup H, Larsen ED, Mollerup S, Tarp H, Soelberg J, Rosthøj S (2020). Long-term follow-up of 60 incarcerated male sexual offenders pharmacologically castrated with a combination of GnRH agonist and cyproterone acetate. Journal of Forensic Psychiatry & Psychology.

[CR27] Cooper AJ (1981). A placebo-controlled trial of the antiandrogen cyproterone acetate in deviant hypersexuality. Comprehensive Psychiatry.

[CR28] Çöpür M, Çöpür S (2021). Chemical castration as an evolving concept: Is it a possible solution for sexual offences?. Journal of Forensic Psychiatry & Psychology.

[CR29] Cortoni F, Marshall WL (2001). Sex as a coping strategy and its relationship to juvenile sexual history and intimacy in sexual offenders. Sexual Abuse.

[CR30] Czerny JP, Briken P, Berner W (2002). Antihormonal treatment of paraphilic patients in German forensic psychiatric clinics. European Psychiatry.

[CR31] Darjee R, Quinn A, Proulx J, Cortoni F, Craig LA, Letourneau EJ (2020). Pharmacological treatment of sexual offenders. The Wiley handbook of what works with sexual offenders: contemporary perspectives in theory, assessment, treatment, and prevention.

[CR32] Day A, Tucker K, Howells K (2004). Coerced offender rehabilitation–a defensible practice?. Psychology, Crime & Law.

[CR33] Douglas T, Bonte P, Focquaert F, Devolder K, Stereckx S (2015). Coercion, incarceration, and chemical castration: An argument from autonomy. Journal of Bioethical Inquiry.

[CR34] Elliott, I. A., & Martin, E. (2023). *Post-release reoffending outcomes for individuals with offence-related sexual paraphilias: An exploratory risk-band analysis.* Retrieved from https://assets.publishing.service.gov.uk/media/63f4954de90e077bb6c6d19a/sexual-paraphilias.pdf.

[CR35] Elliott H, Winder B, Manby E, Edwards H, Lievesley R (2018). “I kind of find that out by accident”: Probation staff experiences of pharmacological treatment for sexual preoccupation and hypersexuality. Journal of Forensic Practice.

[CR36] Finkelhor D (1984). Child sexual abuse: New theory and research.

[CR37] Garcia FD, Thibaut F (2011). Current concepts in the pharmacotherapy of paraphilias. Drugs.

[CR38] Gillespie SM, Mitchell IJ, Fisher D, Beech AR (2012). Treating disturbed emotional regulation in sexual offenders: The potential applications of mindful self-regulation and controlled breathing techniques. Aggression and Violent Behavior.

[CR39] Göbbels S, Ward T, Willis GM (2012). An integrative theory of desistance from sex offending. Aggression and Violent Behavior.

[CR40] Gordon H, Grubin D (2004). Psychiatric aspects of the assessment and treatment of sex offenders. Advances in Psychiatric Treatment.

[CR41] Grubin D (2017). Medication to manage sexual arousal—prescribing guidelines.

[CR42] Grubin D, Beech AR, Carter AJ, Mann RE, Rotshtein P (2018). The pharmacological treatment of sex offenders. The Wiley Blackwell handbook of forensic neuroscience.

[CR43] Guay DRP (2009). Drug treatment of paraphilic and nonparaphilic sexual disorders. Clinical Therapeutics.

[CR44] Hanson RK, Harris AJR (2000). Where should we intervene? Dynamic predictors of sexual offense recidivism. Criminal Justice and Behavior.

[CR46] Hanson, R. K., Harris, A. J. R, Scott, T. -L., & Helmus, L. (2007). *Assessing the risk of sexual offenders on community supervision* (Corrections Research User Report No. 2007–05). Ottawa, ON: Public Safety Canada.

[CR45] Hanson, R. K., & Morton-Bourgon, K. (2004). *Predictors of sexual recidivism: An updated meta-analysis* (Corrections Research User Report No. 2004–02). Ottawa, Ontario, Canada: Public Safety and Emergency Preparedness Canada.

[CR47] Hanson RK, Yates PM (2013). Psychological treatment of sex offenders. Current Psychiatry Reports.

[CR48] Harrison K, Rainey B (2009). Suppressing human rights? A rights-based approach to the use of pharmacotherapy with sex offenders. Legal Studies.

[CR49] Hoffet H (1968). The treatment of sexual delinquents and psychiatric hospital patients with testosterone blocker cyproterone acetate. Praxis.

[CR50] Holoyda BJ, Kellaher DC (2016). The biological treatment of paraphilic disorders: An updated review. Current Psychiatry Reports.

[CR51] Office H (2007). Review of the protection of children from sex offenders.

[CR52] Hughes B (2010). Understanding ‘sexual addiction’ in clinical practice. Procedia Social and Behavioral Sciences.

[CR53] Jeffcoate WJ, Matthews RW, Edwards CRW, Field LH, Besser GM (1980). The effect of cyproterone acetate on serum testosterone, LH, FSH, and prolactin in male sexual offenders. Clinical Endocrinology.

[CR54] Kafka MP (1997). Hypersexual desire in males: An operational definition and clinical implications for males with paraphilias and paraphilia-related disorders. Archives of Sexual Behavior.

[CR55] Khan O, Ferriter M, Huband N, Powney MJ, Dennis JA, Duggan C (2015). Pharmacological interventions for those who have sexually offended or are at risk of offending. Cochrane Database of Systematic Reviews.

[CR56] Knight, R. A., & Thornton, D. (2007). *Evaluating and improving risk assessment schemes for sexual recidivism: A long-term follow-up of convicted sexual offenders* (Document No. 217618). Washington, DC: U.S. Department of Justice.

[CR57] Konrad A, Amelung T, Beier KM (2018). Misuse of child sexual abuse images: Treatment course of a self-identified pedophilic pastor. Journal of Sex & Marital Therapy.

[CR58] Landgren V, Malki K, Bottai M, Arver S, Rahm C (2020). Effect of gonadotropin-releasing hormone antagonist on risk of committing child sexual abuse in men with pedophilic disorder: A randomized clinical trial. JAMA Psychiatry.

[CR59] Landgren V, Savard J, Dhenje C, Jokinen J, Arver S, Seto MC, Rahm C (2022). Pharmacological treatment for pedophilic disorder and compulsive sexual behavior disorder: A review. Drugs.

[CR60] Larkin M, Watts S, Clifton E (2006). Giving voice and making sense in interpretative phenomenological analysis. Qualitative Research in Psychology.

[CR200] Lewis A, Grubin D, Ross CC, Das M (2017). Gonadotropin-releasing hormone agonist treatment for sexual offenders: A systematic review. Journal of Psychopharmacology.

[CR61] Liebling, A. (2012). *Can human beings flourish in prison?* Paper presented at the Prison Phoenix Trust Lecture, London, UK.

[CR62] Lievesley, R. A. (2019). *Navigating MMPSA: Understanding the experiences of individuals convicted of sexual offences taking medication to manage problematic sexual arousal*. Unpublished doctoral thesis, Nottingham Trent University.

[CR63] Lievesley R, Elliott HJ, Winder B, Norman C (2014). Understanding service users’ and therapists’ experiences of pharmacological treatment for sexual preoccupation and/or hypersexuality in incarcerated sex offenders. Journal of Forensic Psychiatry & Psychology.

[CR64] Lievesley R, Winder B, Elliott H, Kaul A, Throne K, Hocken K (2013). The use of medication to treat sexual preoccupation and hypersexuality in sexual offenders. Prison Service Journal.

[CR65] Lincoln YS, Guba EG (1985). Naturalistic inquiry.

[CR66] Lippi G, van Staden PJ (2017). The use of cyproterone acetate in a forensic psychiatric cohort of male sex offenders and its associations with sexual activity and sexual functioning. South African Journal of Psychiatry.

[CR67] Ly T, Fedoroff JP, Briken P (2020). A narrative review of research on clinical responses to the problem of sexual offenses in the last decade. Behavioral Sciences & the Law.

[CR68] Maletzky BM, Field G (2003). The biological treatment of dangerous sexual offenders: A review and preliminary report of the Oregon pilot depo-Provera program. Aggression and Violent Behavior.

[CR69] Maruna S (2001). Making good: How ex-convicts reform and rebuild their lives.

[CR70] Maruna S (2012). Elements of successful desistance signalling. Criminology & Public Policy.

[CR71] Maruna S, Copes H (2005). What have we learned from five decades of neutralization research?. Crime & Justice.

[CR72] Maruna S, LeBel T, Mitchell N, Naples M (2004). Pygmalion in the reintegration process: Desistance from crime through the looking glass. Psychology, Crime and Law.

[CR73] Maruna S, Roy K (2007). Amputation or reconstruction? Notes on the concept of “knifing off” and desistance from crime. Journal of Contemporary Criminal Justice.

[CR74] Medicines and Healthcare Products Regulatory Agency. (2020, June 29). *Cyproterone acetate: new advice to minimise risk of meningioma*. https://www.gov.uk/drug-safety-update/cyproterone-acetate-new-advice-to-minimise-risk-of-meningiomae

[CR75] Meyer WJ, Cole CM (1997). Physical and chemical castration of sex offenders: A review. Journal of Offender Rehabilitation.

[CR76] Nguyen PL, Alibhai SMH, Basaria S, D’Amico AV, Kantoff PW, Keating NL, Penson DF, Rosario DJ, Tombal B, Smith MR (2015). Adverse effects of androgen deprivation therapy and strategies to mitigate them. European Urology.

[CR77] Nnane IP, Worsfold P, Poole C, Townshend A, Miró M (2019). Pharmacokinetics: Absorption, distribution, and elimination. Encyclopedia of analytical science.

[CR78] Nugent B, McNeill F, Furlong A (2017). Young people and desistance. Routledge handbook of youth and young adulthood.

[CR79] Patton MQ (2002). Qualitative research and evaluation methods.

[CR80] Pfaus JG (2009). Pathways of sexual desire. Journal of Sexual Medicine.

[CR81] Prochaska JO, DiClemente CC (1983). Stages and processes of self-change of smoking: Toward an integrative model of change. Journal of Consulting and Clinical Psychology.

[CR82] Ryan RM, Deci EL (2006). Self-regulation and the problem of human autonomy: Does psychology need choice, self-determination, and will?. Journal of Personality.

[CR83] Saleh FM, Grudzinskas AJ, Malin HM, Dwyer RG (2010). The management of sex offenders: Perspectives for psychiatry. Harvard Review of Psychiatry.

[CR84] Sauter J, Stasch J, Klemke K, Emmerling A, Voß T (2018). Discontinuing antiandrogenic treatment in a forensic outpatient setting: A follow-up report of withdrawal trails of a Berlin sample. Forensische Psychiatrie, Psychologie, Kriminologie.

[CR85] Sauter J, Turner D, Briken P, Rettenberger M (2021). Testosterone-lowering medication and its association with recidivism risk in individuals convicted of sexual offenses. Sexual Abuse.

[CR86] Sauter J, Rettenberger M, Briken P, Turner D (2022). Survey on the prescription patterns of pharmacological agents in individuals who have committed sexual offenses during forensic outpatient treatment in Germany: How many discontinue testosterone lowering medication under parole?. Journal of Sexual Medicine.

[CR87] Seebandt G (1968). Thoughts and considerations on the treatment of sex deviation psychopaths with antiandrogens. Das Offentliche Gesundheitswesen.

[CR88] Seligman MEP (1972). Learned helplessness. Annual Review of Medicine.

[CR89] Seto MC (2019). The motivation-facilitation model of sexual offending. Sexual Abuse.

[CR90] Smith JA (2015). Qualitative psychology: A practical guide to research methods.

[CR92] Smith JA, Osborn M, Smith JA (2003). Interpretative phenomenological analysis. Qualitative psychology: A practical guide to research methods.

[CR93] Sowden J, Olver M (2017). Sexual offender treatment readiness, responsivity, and change: Linkages to treatment completion and recidivism. Journal of Forensic Nursing.

[CR94] Sturgess D, Woodhams J, Tonkin M (2016). Treatment engagement from the perspective of the offender: Reasons for noncompletion and completion of treatment—A systematic review. International Journal of Offender Therapy and Comparative Criminology.

[CR95] Thibaut F, Cosyns P, Fedoroff JP, Briken P, Goethals K, Bradford JM, WFSBP Task Force on Paraphilias (2020). The World Federation of Societies of Biological Psychiatry (WFSBP) 2020 guidelines for the pharmacological treatment of paraphilic disorders. World Journal of Biological Psychiatry.

[CR96] Thomas S, Daffern M (2014). Anti-libidinal medication use in people with intellectual disability who sexually offend.

[CR97] Turner D, Basdekis-Jozsa R, Briken P (2013). Prescription of testosterone-lowering medications for sex offender treatment in German forensic-psychiatric institutions. Journal of Sexual Medicine.

[CR98] Turner D, Gregório Hertz G, Sauter J, Briken P, Rettenberger M (2018). Pharmacological treatment of sexual offenders in German outpatient treatment centers. International Clinical Psychopharmacology.

[CR99] Turner D, Petermann J, Harrison K, Krueger R, Briken P (2019). Pharmacological treatment of patients with paraphilic disorders and risk of sexual offending: An international perspective. World Journal of Biological Psychiatry.

[CR100] van den Berg JW, Smid W, Schepers K, Wever E, van Beek D, Janssen E, Gijs L (2018). The predictive properties of dynamic sex offender risk assessment instruments: A meta-analysis. Psychological Assessment.

[CR101] Voß T, Klemke K, Schneider-Njepel V, Kröber HL (2016). If yes, for how long?—Duration of antiandrogenic treatment of sexual offenders with paraphilic disorders. Forensic Psychiatry, Psychology, Criminology.

[CR102] Walton MT, Cantor JM, Bhullar N, Lykins AD (2017). Hypersexuality: A critical review and introduction to the “sexhavior cycle”. Archives of Sexual Behavior.

[CR103] Ward T, Beech AR, Beech AR, Ward T (2017). The integrated theory of sexual offending—revised: A multifield perspective. The Wiley handbook on the theories, assessment and treatment of sexual offending.

[CR104] Ward T, Day A, Howells K, Birgden A (2004). The multifactor offender readiness model. Aggression and Violent Behavior.

[CR105] Ward T, Mann RE, Gannon TA (2007). The good lives model of offender rehabilitation: Clinical implications. Aggression and Violent Behavior.

[CR106] Willig C (2008). Introducing qualitative research in psychology.

[CR107] Winder B, Lievesley R, Elliott H, Hocken K, Faulkner J, Norman C, Kaul A (2018). Evaluation of the use of pharmacological treatment with prisoners experiencing high levels of hypersexual disorder. Journal of Forensic Psychiatry & Psychology.

[CR108] Winder B, Lievesley R, Kaul A, Elliott HJ, Thorne K, Hocken K (2014). Preliminary evaluation of the use of pharmacological treatment with convicted sexual offenders experiencing high levels of sexual preoccupation, hypersexuality and/or sexual compulsivity. Journal of Forensic Psychiatry & Psychology.

[CR109] Wolba J, Tozdan S, Briken P, Freese R, Retz W, Turner D (2023). Changing or stopping testosterone-lowering medication in men convicted of sexual offenses: Clinical evaluation of the COSTLow-R scale. Journal of Sexual Medicine.

[CR110] Yule MA, Brotto LA, Gorzalka BB (2017). Sexual fantasy and masturbation among asexual individuals: An in-depth exploration. Archives of Sexual Behavior.

